# How England got to mandatory biodiversity net gain: A timeline

**DOI:** 10.1007/s13280-025-02277-8

**Published:** 2025-12-03

**Authors:** Alice Stuart, Alan Bond, Aldina M. A. Franco, Chris Gerrard, Julia Baker, Kerry ten Kate, Tom Butterworth, Joseph Bull, Jo Treweek

**Affiliations:** 1 School of Environmental Sciences, University of East Anglia (UEA), Norwich, NR4 7TJ UK; 2 School of Geography and the Environment, Oxford University Centre for the Environment, University of Oxford, South Parks Road, Oxford, OX1 3QY UK; 3 Research Unit for Environmental Sciences and Management, North-West University, Potchefstroom, South Africa; 4 Anglian Water Services Ltd, Lancaster House, Lancaster Way, Ermine Business Park, Huntingdon, Cambridgeshire PE29 6YJ UK; 5 Mott MacDonald, 10 Fleet Place, London, EC4M 7RB UK; 6 Finance Earth (Non-Executive Director), W106, Vox Studios, 1-45 Durham St, London, SE11 5JH UK; 7 RSPB (Trustee), Potton Rd, Sandy, SG19 2DL UK; 8 Arup, 8 Fitzroy St, London, W1T 4BJ UK; 9 Department of Biology, University of Oxford, Oxford, OX1 3SZ UK; 10 eCountability Ltd, Chancery Cottage, Cullompton, Devon EX15 2DS UK

**Keywords:** Biodiversity net gain (BNG), Biodiversity offsetting, Legislation, Net outcome policy, Policy development

## Abstract

Biodiversity net gain (BNG) is a “net outcome” planning policy which aims for development projects to leave biodiversity in a better state than before they started. Understanding the origins and history of existing mandatory BNG is necessary to understand the drivers and barriers that have influenced the policy to date and could inform the development and implementation of future BNG policies. Biodiversity net gain legislation was first discussed in Parliament in England through the passage of the Environment Act (2021) and became a mandatory requirement for most terrestrial and intertidal developments in February 2024. The policy uses habitat attributes as a proxy for biodiversity and represented the widest reaching net outcome policy in the world at the point of its introduction. As such, it is expected to have a significant impact on future land use decisions in England. This paper uses a mixture of literature review and the knowledge of those involved in the early stages of this BNG policy development in England to present a timeline of the stages that have led to mandatory biodiversity net gain. In doing so, we highlight formative events and documents, as an important first step in understanding its history and understanding how this can be used to inform future biodiversity policy.

## Introduction

Net outcome policies are based on a relatively simple premise: that development should aim to achieve an overall “no net loss” or a “net gain” in biodiversity. This extends policy beyond the mitigation hierarchy embedded in Environmental Impact Assessment (EIA) by requiring residual biodiversity losses that are not ecologically irreplaceable to be at least fully compensated for (Bull et al. 2020). This, in theory, allows for continued development while maintaining a neutral or positive overall impact on biodiversity, which is essential if both socioeconomic and ecological targets are to be met (Spaiser et al. 2017; Hickel 2019). In response to this, many governments and organisations have begun to adopt net outcome style policies (Griffiths et al. 2019; zu Ermgassen et al. 2021), with sub-national policies also existing in multiple countries including the UK, Australia, the USA, Canada, and France (zu Ermgassen et al. 2019).

Biodiversity net gain (BNG) is a net outcome planning policy which has a variety of definitions, including developments designed to make their “impact on the environment positive, delivering improvements through habitat creation or enhancement after avoiding or mitigating harm as far as possible” (Defra 2018c, p. 13), and “development that leaves biodiversity in a better state than before. It is also an approach where developers work with local governments, wildlife groups, land owners and other stakeholders in order to support their priorities for nature conservation” (CIEEM, CIRIA and IEMA 2016, p. 2). In England, BNG policy was outlined in the Environment Act (2021) and requires developments within the scope of the policy to demonstrate they will achieve at least a 10% increase in biodiversity units from pre-development before construction can begin. The policy became mandatory on 12 February 2024 (Natural England 2024) for the vast majority of developments falling under the Town and Country Planning Act (1990) (i.e. almost all residential, commercial, and mining related construction) and is anticipated to come into force for Nationally Significant Infrastructure Projects (NSIPs) in late 2025. Given the scope of developments for which BNG is already mandatory and the NSIPs to which it is intended soon to apply, the policy is likely to influence significant decision-making on the use of land both for those undertaking regulated developments and those interested in providing biodiversity units in England.

Documenting the development of BNG in England is an important step in understanding the drivers and constraints that have led to the policy looking as it does today, as well as how this may impact both its implementation in England and the development of future net outcome policies globally. Having a chronicle of formative events and/or policies provides the basis for other researchers, government, and industry professionals to identify the drivers and barriers that can be addressed to support the development of BNG policy elsewhere, as well as understand how to implement future interventions and changes to improve outcomes in England as experience develops. This study, therefore, presents a timeline of the steps leading to the introduction of mandatory BNG in England, representing a first step towards properly understanding its history. In doing so, it collates knowledge of many of the interventions that have established BNG in England and provides a collection of key sources relating to it.

In developing the timeline, it is inevitable that linkages between recent events and the development of current BNG policy are easier to identify compared to those further back in time for which more inferences need to be drawn. To reflect the changing policy landscape, the timeline is divided into seven policy stages:

0. Before 1990, most conservation policies focus on the protection of specific habitats and species, a small number of national offsetting policies arise.
1. From 1990 to 2006, characterised by a global recognition of the need to improve biodiversity outcomes and the inclusion of biodiversity, as opposed to specific protected habitats, in English planning policy, underpinning the future development of specific BNG policy.
2. From 2007 to 2014, characterised by increasing recognition of the value that biodiversity affords human beings, particularly through ecosystem services, in the UK which was reflected in a move to an ecosystems-based approach and exploration of potential biodiversity offsetting legislation in England.
3. From 2014 to 2016, characterised by a more bottom-up approach to the development of BNG approaches and good practice, led by industry.
4. From 2016 to 2019, characterised by Brexit providing the context for the revision of UK environmental protections.
5. From 2019 to 2021, characterised by the passage of the Environment Bill through Parliament, culminating in the adoption of the Environment Act (2021).
6. From 2022 to the time of writing, characterised by preparation for, and the implementation of, mandatory BNG in England.

In presenting this timeline and our interpretation thereof, we record the experience of legislating for net positive biodiversity outcomes, and the associated technical, political and economic issues associated with that. Namely, this process included one of the first national policy impact assessments, if not the first, to quantify significant biodiversity/nature benefits, helping to secure political support for the policy. We describe how economic benefits of strategic ecological restoration are recognised in legislation, modified by the need to demonstrate more immediate developer benefits, demonstrating the tensions within real-world embedding of valuing ecosystem services. We also note some of the practical limitations raised during the process, including available land to realise offsets, limited regulatory authority expertise to assess and monitor outcomes, and long-term responsibility for outcomes.

## Methods

This timeline has been produced in two stages. Initially, a broad timeline was produced using the information available in key documents and government reports on BNG found through previous research on BNG, mainly regarding the initial 2018 Defra consultation on net gain (e.g. Defra 2018a, b, c) and related documents. These sources were then supplemented by taking a snowballing approach, following references from the identified sources and investigating events and reports mentioned in any relevant literature. The dates of any events and documents directly relevant to BNG in England were recorded in a table and a note was made of their relevance, primarily consisting of changes to legislation, mention of future dates and events, or approach to BNG that were mentioned within the documents. At this point, the timeline was split into the six sections between “from 1990 to 2006” and “from 2022 to the time of writing” presented here, both to increase the readability of the document, and to highlight perceived shifts in approach to biodiversity leading to mandatory BNG in England. A summary paragraph was written for each section of the timeline to allow the reader to quickly determine relevance without the need to read the detail of every event. The references for all events within these summary paragraphs are contained within the tables below, with key events related to the summary texts underlined.

After developing an initial understanding, consultation was undertaken with academics and practitioners involved in BNG in England. This approach helped to identify additional drivers, events, and interpretations not well documented in the literature, and also additional people to consult. It was during this stage that the pre-1990 section was added in recognition of the importance of early international policies that set the context for BNG in England, and this was therefore referenced as stage “zero”, creating seven stages in total. In addition, international context was added to the summary paragraphs at the start of each timeline section where relevant. All people consulted have had their contribution acknowledged, either through authorship or within the acknowledgements section. Where information has been included based on the personal knowledge and experience of those involved in the policy evolution, as opposed to a more referenceable source, it has been highlighted in italics to make the provenance clear.

## Timeline

**Fig. 1:**
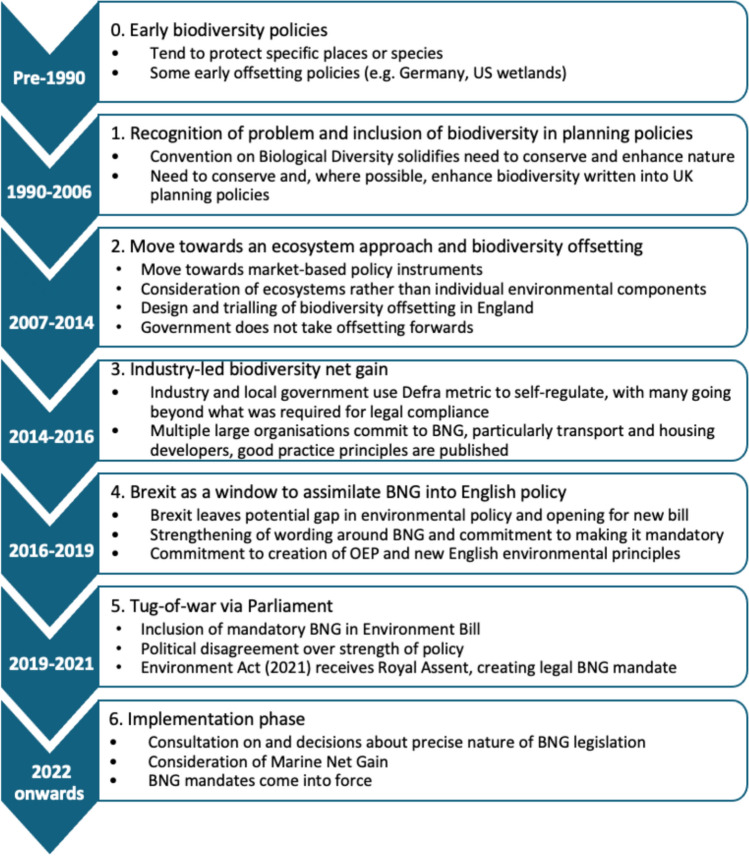
Summary of stages of English BNG policy development. Note that the OEP (Office for Environmental Protection) was set up in November 2021 to protect and improve the environment of England and Northern Ireland (and reserved matters for the UK), by holding government and other public authorities to account post-Brexit

An overview of the stages involved in the development of English BNG policy are shown in Fig. 1.

### 0) Pre-1990: Early biodiversity policies

Early biodiversity policies focussed on specific places and landscapes, for example the Yellowstone National Park Act (1872), considered to be the first case of an area being formally protected in law with a primary purpose of preserving nature (U.S. National Park Service 2020) and, in the UK, the protection of designated areas, initially through the National Parks and Access to the Countryside Act 1949. Later came policies designed to protect species, such as the Clean Water Act in 1972 (Hines 2012) and Endangered Species Act in 1973 in the USA, as well as the Birds Directive in 1979 (European Commission 2024) in the EU. Subsequently, in recognition of the extent to which development is a leading cause of biodiversity loss, multiple countries brought in offsetting-style policies; including Germany, which introduced national mandatory biodiversity offsetting in 1976 (Tucker 2016), and the US, where no net loss was suggested as a goal for US wetlands policy at the National Wetlands Policy Forum in 1987 and adopted into policy in 1989 (Heimlich et al. 1998).

#### Stage 1 (1990 to 2006): Biodiversity enters planning UK policy

During this period, there is increasing concern about the implications of continued biodiversity loss and the need to halt and, where possible, reverse this. Following the adoption of the Convention on Biological Diversity in 1992 (United Nations 1992), the UK adopted a biodiversity action plan and considered using the planning system to minimise harm caused by development and, where possible, use it to enhance biodiversity. Elsewhere, no net loss continued to be adopted as a biodiversity policy, for example, in the states of New South Wales, Victoria and Western Australia in Australia (ten Kate et al. 2004 Box 12). Also during this time, interest in offsetting within the private sector increased (e.g. ten Kate et al. 2004) leading to the founding and first meeting of the Business and Biodiversity Offsets Programme in 2004 (BBOP 2018). Key events in England during this period are shown in Table 1.

**Table 1:** Key events of Stage 1 (1992–2006) in which biodiversity enters UK planning policy

Year	Month	Event	Relevance to BNG
1990	November	Environmental Protection Act 1990 (Environmental Protection Act 1990)	• Amongst other things, split the Nature Conservancy Council, up until that point responsible for designating and managing National Nature Reserves and other nature conservation areas in the UK into three national bodies and the Joint Nature Conservation Committee • The English body became known as English Nature, later to become Natural England
1992	May	Convention on Biological Diversity (United Nations 1992)	• Recognised need for nations to conserve and enhance biodiversity • Identified need for global scientific ecosystem assessment • UK sign up, committing to conserve and protect existing biological diversity, and to enhance it wherever possible, including drawing up a national biodiversity action plan
1994	January	UK Biodiversity Action Plan published (Department of the Environment 1994)	• Required by Convention on Biological Diversity (1992) • Recognition of need “to ensure the conservation and, where possible, the enhancement of biodiversity within the UK” (p. 3) • Set priority species and habitats
1995	July	The Environment Act 1995 (Environment Act 1995)	• Created some provisions for “the conservation of natural resources and the conservation or enhancement of the environment” (p.1) amongst other things
2000	April	UN announce The Millennium Ecosystem Assessment (Annan 2000)	• Announced by UN Secretary-General Kofi A. Annan • Intended to provide scientific evidence for future policy
	May	COP-5 adopts the ecosystem approach and defines principles for its use (United Nations 2000)	• Defines the ecosystem approach as “a strategy for the integrated management of land, water and living resources that promotes conservation and sustainable use in an equitable way” (Annex A) • Makes a call for governments and organisations to use the ecosystem approach as appropriate • Makes it clear that the “ecosystem approach does not preclude other management and conservation approaches” (Annex A) • Provides principles for the use of the ecosystem approach (Annex B)
	November	Countryside and Rights of Way Act (Countryside and Rights of Way Act 2000)	• Required the Minister of the Crown, Government departments, and the National Assembly for Wales “to have regard … to the purpose of conserving biological diversity in accordance with the Convention [on Biological Diversity of 1992]” (Part III, Sect. 74.1) • Created duty to publish lists of habitats and species of principle importance and take and promote “reasonably practicable” steps “to further the conservation of the living organisms and types of habitat included in [said lists]” (Part III, Sect. 74.3)
2002	October	Defra publish “Working with the grain of nature” a new biodiversity strategy for England (Defra 2002)	• Set the aim to “ensure that construction, planning, development and regeneration have minimal adverse impacts on biodiversity and enhance it where possible” (p. 53) • Suggests action towards “[p]lanning policies and development decisions that recognise the need to conserve and enhance biodiversity” (p. 57)
2004	December	IEEM Fellows lecture putting forwards mitigation banking in England (Hill 2004)	• Pushed for mitigation banking to be investigated as an approach for conservation in the UK • Suggested environmental stewardship schemes could be linked with mitigation banking, “enabling greater biodiversity gains” (p. 6)
2005	January	Planning Policy Statement 1: Delivering Sustainable Development (Office of the Deputy Prime Minister 2005a)	• Set out that planning authorities “should seek to enhance the environment as part of development proposals” (para. 19) • Stated that “[i]n line with the UK sustainable development strategy, environmental costs should fall on those who impose them—the “polluter pays” principle” (p. 19)
	March	UN Millennium Ecosystem Assessment (MEA) published (Millennium Ecosystem Assessment 2005)	• Influenced thinking in the UK, leading to the UK NEA (Waylen and Young 2014)
	August	Planning Policy Statement 9: Biodiversity and Geological Conservation (Office of the Deputy Prime Minister 2005b)	• Included ensuring that biodiversity is conserved and enhanced as “an integral part” of development as a key Government objective for planning (Page 2) • Reiterated that “Plan policies and planning decisions should aim to maintain, and enhance, restore or add to biodiversity”
2006	March	Natural Environment and Rural Communities Act (Natural Environment and Rural Communities Act 2006)	• Creates more general duty to conserve biodiversity (Sect. 40), updating that previously set out in the (Countryside and Rights of Way Act 2000), to require that “[e]very public authority must, in exercising its functions, have regard, so far as is consistent with the proper exercise of those functions, to the purpose of conserving biodiversity” (p. 14) • Established Natural England by merging English Nature, the Rural Development Agency and the Countryside Agency
	September	The Environment Bank Ltd is incorporated (Companies House 2024)	• *Established to lobby for, and undertake, biodiversity offsetting projects*

#### Stage 2 (2007–2014): Nature as offset-able ecosystems

*This period saw a move towards spatial planning for biodiversity outcomes and recognition of the need to address losses of biodiversity in the wider landscape, rather than focusing only on designated and protected sites, habitats and species.* Throughout this period, there was an emphasis on the need for companies to internalise their environmental impact *associated with substantial internal lobbying for biodiversity offsetting within Natural England* and the government commission significant amounts of research on ecosystems, biodiversity offsetting and biodiversity markets. Biodiversity offsetting is scoped and trialled as a policy option in England to see if it could more efficiently and effectively deliver existing biodiversity planning and consent processes, accompanied by a political push for market-based conservation methods. The UK government introduce a no net loss objective and net gain aim. During this period, considerable information exchange occurs between the UK policy makers and other countries with established offsetting policies through conferences *and meetings*. Media push-back occurs against offsetting as a policy, considering it a “licence to trash”.

*Independently to the development of biodiversity offsetting*, there is the publication of a national assessment of UK and English ecosystems, the state they are in, and the economic value they confer, as well as a review of the effectiveness of current nature conservation sites, also prompted interest in ecosystem approaches. Elsewhere, other countries continue to adopt net outcome policies, notably in Europe, with the European Parliament calling for No Net Loss regulation using BBOP standards in 2012 (BBOP 2018); the European Commission consultation on no net loss in 2014 (European Commission 2024); and France introducing NNL into guidance developed in 2012/13, and into law in 2016 (Vaissière et al. 2018). Key events in England during this period are shown in Table 2.

**Table 2:** Key events of Stage 2 (2007–2014) in which nature is increasingly seen as offset-able ecosystems

Year	Month	Event	Relevance to BNG
2007	January	House of Commons Environmental Audit Committee review the MEA (Environmental Audit Committee 2007b)	• Reiterates need for companies to internalise their environmental impact • Recommend that the government assess UK ecosystems to identify and develop effective policy responses (para. 30)
	June	UK Species and Habitat Review concludes (Biodiversity Reporting and Information Group (BRIG) 2007)	• Updated UK BAP priority species and habitats
	July	Government response to Environmental Audit Committee’s review of MEA (Environmental Audit Committee 2007a)	• Early mention of the need for metrics for ecosystem services to aid in internalising business externalities (p. 6) • References the upcoming Defra Ecosystems Approach Action Plan as a solution to better valuation of ecosystem services (p. 13) • References that work on “status and trends in England’s terrestrial ecosystems, and the goods and services they provide” (p. 17) is being done
	October	Defra and UK Biodiversity Partnership publish Conserving Biodiversity—The UK Approach (Defra and UK Biodiversity Partnership 2007)	• Designed to provide a strategic framework for conserving biodiversity in the UK in the light of changing pressures and increasing devolution • Pushes the importance of the ecosystem approach as decided in COP-5 (United Nations 2000) • Discusses the importance of targeting action to priority species and habitats and embedding “proper consideration of biodiversity and ecosystem services into all relevant sectors of policy and decision-making” (p.10) • Identified a need to explore new policy options for ecosystem conservation, possibly including the creation of a market in biodiversity or new incentives for biodiversity “such as biodiversity offsets”, particularly to reduce the loss of non-designated sites and features (Treweek 2009)
	December	Defra publish Ecosystems Approach Action Plan (Defra 2007)	• Cohesive ecosystems-based approach rather than considering environmental elements in separate policies
2008	*Early*	Results of Defra-commissioned scoping study for UK MEA-style ecosystem assessment published (Haines-Young et al. 2008)	• Suggests that it would be possible and would provide benefits but may be too expensive if not mainly built based on existing research
	*Unknown*	Defra commission a scoping study for the design and use of biodiversity offsets in an English context (Treweek 2009)	• Sought to use offsetting fulfil duties under the Countryside and Rights of Way Act (2000), the Natural Environment and Rural Communities Act (2006) and associated planning policy • Identifies how offsets could be set up in the UK and how this would fit with current legislation
2009	*Unknown*	BBOP Principles, Handbooks, Resource Papers, Glossary and Case Studies published (BBOP, 2009a, 2009b)	• Marked completion of Phase I of BBOP’s work • Provided an international best practice for biodiversity offsetting • Suggested the use of different metrics (inc. area based; area x quality; species density and occupancy) depending on context • Principles state that projects using offsets should follow the mitigation hierarchy, recognise that some biodiversity is irreplaceable, ensure offsets result in both additional conservation outcomes that are secured for at least the lifetime of the project and equitable social outcomes based on stakeholder engagement, and both science and traditional knowledge
	April	Results of English offsetting scoping study published (Treweek 2009)	• Found that “biodiversity offsets are unlikely to be implemented to any great extent under current EU law and associated regulations” (p. 3) • Suggested more consideration of whether new regulation would be required to ensure a regular and consistent “no net loss of biodiversity” requirement for development and systems for trading biodiversity credits • Suggested need for a series of pilot projects • Put forward a habitat-based metric calculating units as *area (ha) x distinctiveness x condition*, later used in the 2012 Defra offsetting pilots
	mid-year	UK National Ecosystem Assessment commences as part of the Living With Environmental Change (LWEC) initiative (UNEP WCMC 2009)	• Was expected and initiated to produce evidence that could be used to inform future policy (Waylen and Young 2014)
	Sept	Lawton Review commissioned (Lawton et al. 2010, p. ii)	• Commissioned by Hilary Benn, the then Secretary of State in the Department for Environment, Food and Rural Affairs, to review whether England’s wildlife sites were capable of adapting to climate change and other land uses
2010	January	Possible methods for measuring biodiversity losses and gains for use in the UK published (Treweek et al. 2010)	• Requires an ecosystem approach to value areas as a whole rather than their individual components • Recommended a minimum of 1:1 ratio of area of compensation to area of habitat lost • Recognised that some important attributes would not be captured by a habitat-based system
	April	Conservative party release election manifesto (Conservative Party 2010)	• Discusses a move away from “rules and regulations to impose a centralised worldview” to “new incentives and market signals” (p. 89) • Includes proposal for the increasing the “market for green goods and services” (p. 89) and “a new system of conservation credits to protect habitats” (p. 96)
	May	UK general election results in a Conservative-Liberal Democrat coalition (Rhodes et al. 2011)	• Conservatives win the most seats but not a parliamentary majority • Allows Conservatives to begin enacting their proposed environmental policies
	July	Defra publish discussion document in advance of 2011 White Paper (Defra 2010a)	• Suggests biodiversity offsetting to increase the role of “Big Society”, as opposed to “Big Government”, in ensuring sustainable natural resource use
	September	Lawton review published (Lawton et al. 2010)	• Suggested four main principles for improvement: bigger, better, more, and joined up • Suggested the need for considerable leadership from government • Set out principles for effective biodiversity offsetting
	December	Defra post discussion materials about biodiversity offsetting on website (Defra 2010b)	• Intended to feed into the 2011 Natural Environment White Paper • Suggested using Sect. 106 (dealing with planning obligations in the Town and Country Planning Act 1990) payments for offsetting • Summary of responses, published in July 2011 (Defra 2011a) showed respondents were broadly positive • Concerns about the potential for offsetting to undermine the mitigation hierarchy, increased burden including expertise requirements in local authorities, and implications of maintaining offsets “in perpetuity”
2011	January	Biodiversity Offsetting POSTnote published (POST 2011)	• Provided a summary of biodiversity offsetting for members of Parliament
	May	Defra publish 2011–2015 business plan (Defra 2011b)	• Had “Assess the scope for actions to offset the impact of development on biodiversity” as an action point
	June	UK National Ecosystem Assessment published (UK National Ecosystem Assessment 2011)	• Identified land use change as a major factor in ecosystem declines and suggested offsetting as one part of the solution (UK National Ecosystem Assessment 2011) • Provided much of the evidence for the government white paper (Watson 2012) however, this was due to contact between departments, not the original intention (Waylen and Young 2014)
		UK Government White Paper “The Natural Choice: securing the value of nature” (Defra 2011c)	• Promoted the importance of markets for ecosystem services (p. 4) • Set a no net loss objective with plan to move to net gain • Emphasised the role of planning in securing a sustainable future, but lamented the costly and bureaucratic nature of existing systems (para. 2.33–2.34) • Discussed the upcoming National Planning Policy Framework (NPPF) as a solution to planning issues, including a “new presumption in favour of sustainable development” (para. 2.37) • Introduced biodiversity offsetting as a means of allowing development to achieve no net loss, based on the principles set out in the Lawton Review (para. 2.38–2.40) • Introduced the plan for a two-year offsetting pilot testing a new voluntary approach in certain local authorities, running from Spring 2012 (para. 2.41) • Committed to setting up a business-led Ecosystem Markets Task Force to report “to review the opportunities for UK business from expanding green goods, services, products, investment vehicles and markets which value and protect nature’s services” (Annex I: para. 44) reporting back in 2013
2012	Jan-June	BBOP Standard, Guidelines, and more Resource Papers published (BBOP 2012b, 2012a, 2012c)	• The result of BBOP’s Phase II work • Included a published standard for biodiversity offsets and new guidance for measuring losses and gains
	March	National Planning Policy Framework (NPPF) published (Department for Communities and Local Government 2012)	• Substantially simplified the planning process, replacing 44 pieces of previous planning policy guidance • First use of “net gain” with respect to biodiversity in English planning policy, stating that “[t]he planning system should contribute to and enhance the natural and local environment by … minimising impacts on biodiversity and providing net gains in biodiversity where possible” (Department for Communities and Local Government, 2012, para. 109) • Provided a legislative justification for local councils to aim for net gain
	April	Two-year offsetting pilots begin (Defra and Natural England 2012)	• Aimed to assess whether biodiversity offsets helped to streamline planning process and deliver greater benefits for biodiversity (Baker et al. 2014) • Included six pilot local planning authorities: Doncaster, Devon, Essex, Greater Norwich, Nottinghamshire or Warwickshire with Coventry and Solihull • Guidance for using the habitat metric put forward in Treweek, Butcher and Temple (2010) (p. 5–7), did not include a minimum compensation, *although the pilots were expressly designed not to test the metric* • First English guidance for offset requirements (broadly like-for-like or better; p. 8) • Emphasised importance of the mitigation hierarchy (p. 4) • Allowed organisations to provide their own offsets or purchase them from a provider
	July	UK BAP succeeded by UK Post-2010 Biodiversity Framework (JNCC and Defra 2012)	• Introduces targets that “[b]y 2020, at the latest, biodiversity values have been integrated into national and local development” and “positive incentives for the conservation and sustainable use of biodiversity are developed and applied” (p. 11)
	Onwards	Early news coverage against biodiversity offsetting (Monbiot 2012)	• Describes offsetting as a "licence to destroy"
2013	Unknown	The Thameslink Programme voluntarily set target to achieve BNG for the second phase of the Thameslink upgrade (Defra 2013a)	• Very early adopter of BNG
	February	POSTnote on potential solutions for biodiversity and planning decisions published (POST 2013)	• Summarises potential policies that might improve the planning system to address biodiversity loss • Discusses biodiversity offsetting for compensation
	March	Final Report of the Ecosystem Markets Task Force published (Ecosystem Markets Task Force 2013)	• Includes mandating biodiversity offsetting as the number one priority recommendation for the government • Sees biodiversity offsetting as a way to save developers time and money, revolutionise conservation in England, and stimulate the competitive growth of businesses
	May	Defra summit on biodiversity offsetting (British Ecological Society 2013)	• Called by Owen Paterson, the Secretary of State for the Environment, Food and Rural Affairs • Patterson discussed his trips to understand the Australian systems and reported general cabinet support for biodiversity offsetting
	September	Government respond to Ecosystem Markets Task Force report (Defra 2013b)	• Announce green paper consultation on biodiversity offsetting • Emphasise that “an offsetting system must deliver benefits for development” (p. 7) and suggest a permissive approach “giving developers the choice to use biodiversity offsetting where it would enable them to meet existing requirements more efficiently than happens currently” (p. 7) • Stated that “Following the Green Paper consultation the Government will develop its detailed proposals for using biodiversity offsetting and plans to set these out by the end of 2013” (p. 7)
		Meeting of experts promoting species considerations for biodiversity offsets in England (Howard and Gent 2013)	• Highlighted "need to designate a set of approaches to offsetting for impacts on each species requiring special consideration in biodiversity offsets" (p. 1) • Came up with recommendations as to how species should be considered going forwards, including coming up with a list of priority species and further evidence collection as to habitat suitability
	Autumn	National Grid state voluntary aim to create biodiversity gain (National Grid 2013)	• “National Grid aims to create biodiversity gains by using its land to create a natural grid of better and bigger habitats” (p. 6)
	Sept-Nov	Defra Green Paper consultation on introducing biodiversity offsetting in England (Defra 2013a)	• Presented offsetting as a means to tackle the “twin challenges of growing its economy and improving its natural environment” (both p. 1) as well as reducing uncertainty and cost in development and planning • Stated the Government would only bring in an offsetting system if it would make the planning system related to biodiversity “quicker, cheaper and more certain for developers”; “[a]chieve net gain for biodiversity” by ensuring no net reduction in number of units “and seeking to locate offsets in a way that enhances ecological networks (achieving “net gain”)”; and “[a]void additional costs to businesses” (all p. 8) • Results published in February 2016 (Defra 2016) found a slim majority (53%) of respondents wanted offsetting • The majority of respondents from the public opposed offsetting, either in principle or due to a lack of confidence in the proposed system
		Consultation triggers new wave of negative press (e.g. Carrington 2013; Howarth 2013; Stringer 2013)	• Continue to present offsetting as “a licence to trash nature”
	Oct-Nov	Environmental Audit Committee biodiversity offsetting enquiry (Environmental Audit Committee 2013)	• Launched to look into the Government consultation on biodiversity offsetting in England • Reported that offsetting should only be brought in if, after the pilots had been completed and independently assessed, offsetting was found to bring benefits • Considered the metric too simplistic and that a “proper metric needs to reflect the full complexity of habitats, including particular species and “ecosystem networks”, and recognise the special status of ancient woodlands and sites of special scientific interest” (p. 3) • Emphasised the need to follow the mitigation hierarchy and for offsets to be “near enough to the local development that local people can still enjoy [them]” (p. 3) • Stated if biodiversity offsetting were to be brought in, it would need to be mandatory
	Nov	HS2 publish biodiversity metric and set route-wide NNL target (Department for Transport and High Speed Two (HS2) Limited 2013)	• Broadly similar to Defra metric but first included irreplaceable habitats (which were later removed) and had shorter time to target condition (Natural England 2016c)
2014	March	Report to Defra on lessons learnt from biodiversity offsetting markets in other countries (Duke and ten Kate 2014)	• Designed to gather evidence from the US and Australia (existing offsetting markets) • Found benefits for developers including efficiency, unblocking developments and reduction in liabilities • Found market design greatly impacts cost and availability of units • Found on-site compensation delivers poor conservation outcomes • Found considerable economic benefit from market and speeding up development
	April	Offsetting pilots end (Baker et al. 2014)	• Involved stakeholders generally felt that Defra metric v1 was a consistent, transparent and simple method to measure biodiversity changes that accounted for a wider range of impacts than prior practice • Some stakeholders had concerns that the metric omitted certain ecological aspects, was more intensive than current practice, and misvalued certain habitat types • All but one of the pilots felt that a voluntary system was insufficient to support widespread biodiversity offsetting • In some cases, offsetting was presented by developers as a means to compensate for, instead of avoid, damage potentially undermining the mitigation hierarchy • Many developers challenged the increased compensation requirement identified by the metric • Found that the current system was not meeting no net loss as measured by the metric • Concluded that offsetting had the potential to provide improved biodiversity outcomes if additional resources were provided to fund ecological expertise in local authorities but that it would result in increased costs to developers and the benefits in terms of streamlining the planning process were, at best, marginal • Publication of metric allowed other organisations to take it on and use it
	June	“To No Net Loss of Biodiversity and Beyond” conference co-hosted by Forest Trends, BBOP, ZSL and Defra in London (Forest Trends et al. 2014)	• Included 280 individuals from 32 countries • Hosted by Forest Trends, the Business and Biodiversity Offsets Programme (BBOP), the UK Department for Environment, Food and Rural Affairs (Defra), and the Zoological Society of London (ZSL) • Identified need for clear policy for no net loss or BNG to become a reality as well as needs to build capacity, strengthen protection, ensure monitoring and enforcement, and consistently apply mitigation hierarchy
		2nd Forum of Natural Commons held in Regent’s Park Hub, London (Verpoest 2014)	• Held to protest no net loss conference • Panels on the narrative behind valuing nature and the potential impact of biodiversity offsetting on communities

#### Stage 3 (2014–2016): Industry led BNG

The government do not take offsetting forward, *anecdotally due to the negative press and reaction to pilot projects combined with the removal of Owen Paterson, a major proponent of offsetting as an approach, from cabinet*. Meanwhile, *many* of the local planning authorities involved in the offsetting pilots continue with offsetting on a voluntary basis. Industry takes the tools published for the offsetting pilots to set and demonstrate progress towards voluntary targets of NNL and BNG that go beyond compliance *and help to shift attitudes in industry to move from ecology being an issue of risks to a measurable sustainability opportunity*. This, *combined with individuals within organisations pushing for better biodiversity outcomes*, leads to multiple projects piloting a BNG approach and multiple industry and governmental organisations committing to BNG. The good practice guidelines are put together *building on the international principles published by BBOP* and published in response to the need to bring some standardisation to practice and to set out good practice. Local government and industry began calling for mandatory BNG to further standardise practice and provide a “level playing field”. Key events in England during this period are shown in Table 3.

**Table 3:** Key events of stage 3 (2014–2016) in industry lead progress towards BNG

Year	Month	Event	Relevance to BNG
2014	July	Owen Paterson, Environmental Secretary and major proponent of offsetting, loses position in cabinet reshuffle (Phipps 2014)	• *Potentially related to government decision not to take offsetting forward*
	*Unknown*	Transport for London publish framework (Butterworth et al. 2019, p. 30)	• Includes aim to “protect, manage and enhance the natural environment within our land holding” (Butterworth et al. 2019, p. 30)
2015	January	Warwick District submit local plan including BNG aim (Warwick District Council 2015)	• States that “the Council seeks to protect the natural environment and strives for net gains in biodiversity” (p. 154) • Plan adopted in 2017 (Council 2024) • Approach to BNG and offsetting included the development and use of a “locally derived Defra metric” (Lowe 2019)
	March	Department for Transport publish Road Investment Strategy: for the 2015/16–2019/20 Road Period (Department for Transport 2015)	• Includes aspiration for NNL by 2020 and BNG by 2040
	June	Highways England publish biodiversity plan (Highways England 2015)	• Reiterates plan for roads to achieve BNG by 2040 • Includes commitment to creating or adopting a biodiversity metric by December 2017
	October	Barratt Homes include habitat enhancement in operational principles (Barratt Developments plc 2015)	• State that they “seek to enhance habitats, biodiversity and local environments across all of our developments”. • Early steps towards BNG in housing sector
	(pre-)November	Network Rail Infrastructure pilot Projects make commitment for net positive for biodiversity to be business-as-usual by March 2019 (Darbi 2015; IEMA 2015)	• A series of webinars discuss Network Rail Infrastructure Projects’ commitment to achieving a “measurable net positive contribution towards biodiversity in the UK” (Darbi 2015) • And “plans for Net Positive to become business-as-usual by March 2019” (IEMA 2015)
	December	Lichfield District Council introduce BNG aim (Lichfield District Council 2015)	• “Core Policy 13: Our Natural Resources is the over arching policy which… seeks to deliver a net gain for biodiversity where impacts arise from development proposals” (p. 31)
2016	February	Defra publish summary of responses to 2013 Green paper biodiversity offsetting consultation (Defra 2016)	• Next steps section does not discuss taking offsetting forwards, instead stating they will “continue to work … to further our shared understanding of how best to compensate for biodiversity loss when it cannot first be avoided or mitigated” (p. 37)
	May	Lichfield District Council introduce BNG requirement (Lichfield District Council 2016a, b)	• “Developments which take into account the role and value of biodiversity … and must deliver a net gain for Biodiversity” (p. 6)
	October	Industry increasingly adopt BNG	• WSP publish report on BNG and its role in infrastructure (WSP and Parsons Brinckerhoff 2016), predicting BNG’s inclusion in planning policy and discussing the usefulness of creating a consistent understanding to create a level playing field for developers • Crossrail 2 introduce BNG aim • Barratt Homes introduce a net positive biodiversity target (Barratt Developments plc 2016)
	November	“Enhancing Natural Capital and Delivering Biodiversity Gain Through Planning and Development” Conference (Natural England 2016a, b)	• Convened by Natural England and hosted by National Rail • Considerable discussion with many partner organisations on moving from offsetting to net gain and how to deliver net positive • *Results in Natural England’s renewed involvement in what became known as BNG and the organisational commitment to then try to take forward work on standards and metric updates*
	December	Biodiversity Net Gain: Good Practice Principles for Development published (CIEEM, CIRIA and IEMA 2016)	• Industry led principles for good practice BNG that contributes to strategic priorities and sustainable development adapted from the BBOP principles • Gave industry criteria to show that projects have followed good practice • Included a clear definition of BNG as “development that leaves biodiversity in a better state than before. It is also an approach where developers work with local governments, wildlife groups, landowners and other stakeholders in order to support their priorities for nature conservation” (p. 2)

#### Stage 4 (2016–2019): Brexit policy shock

The UK votes to leave the EU, meaning the 80% of UK environmental legislation derived from the EU is up for debate (Friends of the Earth 2021) *creating a window for substantial environmental policy change* and catalysing the passage of the Environment Bill through Parliament. BNG is proposed for inclusion in the Environment Bill *and is perceived by some as a means of financing manifesto commitments to environmental improvement in the face of austerity and fiscal restraint.* The government consults on making BNG mandatory, leading to a commitment to include it in the Bill. Requirements for environmental legislation post-Brexit are negotiated between the House of Commons and House of Lords. Independently of Brexit, the UK government moves towards biodiversity net gain consideration, including the first policy mention of “measurable” BNG. Net outcomes continue to increase in popularity within industry and, by 2018, some 60 companies worldwide were estimated to have public, company-wide commitments or aspirations for No Net Loss of biodiversity or similar (BBOP 2018). Key events in England during this period are shown in Table 4.

**Table 4:** Key events in Stage 4 (2016–2019) in which Brexit creates a policy shock and opens a window of opportunity for BNG

Year	Month	Event	Relevance to BNG
2016	June	UK referendum on UK membership of the EU results in 3.8% winning margin for leave, subsequently known as Brexit (Uberoi 2016)	• 80% of the UK’s environmental laws at the time derive from the EU (Friends of the Earth 2021), potential for all of these to be changed • Societal will for stronger environmental legislation: in a survey, 83% of people surveyed said Britain’s new environmental laws after Brexit should be at least as good (37%) or even better (46%) than those from the EU (Carrington 2016)
2017	March	European Union (Notification of Withdrawal) receives Royal Assent (European Union (Notification of Withdrawal) Act 2017 (c. 9), 2017)	• Set the legislative process of Brexit in motion
	May	Berkeley Group commit to achieve biodiversity net gain on new developments (Berkely Group 2017)	• The “first developer [in England] to commit to achieving a net biodiversity gain on every new site” (p. 9)
	July	First reading of the European Union (Withdrawal) Bill (Department for Exiting the European Union 2018)	• First public version of the legal requirements for the UK after leaving the EU • Little discussion of environmental issues (HM Parliament 2018)
	August	Mayor of London publishes draft London Environmental Strategy (Mayor of London 2017)	• Includes policy 5.2.1 to “[p]rotect a core network of nature conservation sites and ensure a net gain in biodiversity” (p. 161)
2018	*Unknown*	BBOP publish Roadmaps for Government and Business, Resource Papers, and Overview with Call to Action (BBOP 2018)	• Marked the conclusion of BBOP’s activities • Provided clear and actionable roadmap and guidance for governments and businesses wanting to go forwards with offsetting • Aimed at following the mitigation hierarchy to achieve at least No Net Loss and preferably a Net Gain
	January	UK Government publish 25 Year Environmental Plan “A Green Future: Our 25 Year Plan to Improve the Environment” (HM Government 2018)	• Commits to ambitious development targets and to “embed a ‘net environmental gain’ principle for development … enabl[ing] housing development without increasing overall burdens on developers” (p. 33) • *BNG had been identified by Michael Gove, the Secretary of State for Environment, Food and Rural Affairs, as an opportunity to improve nature development outcomes, providing important political weight to the policy* • Committed to “[m]aking sure that existing requirements for net gain for biodiversity in national planning policy are strengthened, including consulting on whether they should be mandated alongside any exemptions that may be necessary” (p. 34)
	Feb-May	European Union (Withdrawal) Act debated in the House of Lords (Maer 2018a)	• Non-government amendment requiring the protection of EU environmental principles and standards, including equivalent independent oversight, added on the third reading
	May-Aug	Defra launch Consultation on Environmental Principles and Governance after EU Exit (Defra 2018b)	• Set out that a statutory policy statement on principles and accountability, including the creation of a new body to hold government to account, would be created through an Environmental Principles and Governance Bill • Appeared to move towards environmental net gain, causing some concerns (e.g. Environmental Audit Committee 2018a, para. 139) leading the Government to clarify that “biodiversity net gain is, and should remain, the central pillar around which wider approaches might be developed” (Environmental Audit Committee 2018b, p. 16) and that “developing the concept of environmental net gain will take place over a longer timescale” (Environmental Audit Committee 2018b, p. 17)
	June	European Union (Withdrawal) Act returns to House of Commons (Maer 2018b)	• Lords’ amendment requiring protection of EU environmental standards voted against and replaced with weaker obligation for the Government to publish environmental principles within six months of the bill and to make provisions for the creation of a public body able to take enforcement action against the government
		European Union (Withdrawal) Act receives Royal Assent	• New amendments from the House of Commons unchallenged • Set legal requirements to publish environmental principles and make provisions for a new public body for enforcement
	July	National Planning Policy Framework revised (Ministry of Housing, Communities and Local Government 2018)	• Strengthens wording around BNG (“should” rather than “where possible”, adds “measurable”): “plans should … identify and pursue opportunities for securing measurable net gains for biodiversity” (para. 174)
	November	EU-UK withdrawal agreement (with backstop) (House of Commons Library 2019)	• Required non-regression from EU environmental standards after Brexit to avoid a hard border between Northern Ireland and Ireland if the Northern Ireland protocol were triggered
		Natural England post about development and trialling of updated metric (Natural England 2018)	• Promised improved treatment of ecological connectivity, greater habitat type coverage, and a new spreadsheet-based tool for application
	December	Government Publish draft version of Environment (Principles and Governance) Bill (Defra 2018a)	• Met legal requirements set by the European Union (Withdrawal) Act (2018) to publish environmental principles and make provisions for a new public body, the Office for Environmental Protection (OEP) intended to replace the role of the EU Commission post-Brexit in ensuring regulatory compliance with EU derived environmental legislation • Concern that other parts of the bill, including BNG, had not been submitted for scrutiny (Environmental Audit Committee 2019)
2018–2019	Throughout	Multiple organisations adopt biodiversity net gain and develop biodiversity metrics	• “Network Rail Biodiversity Calculator” (Network Rail 2018) • Highways England “biodiversity metric” (Highways England 2019b, a) • Transport for London “toolkit” (Jackman 2019) • SSE “Full BNG Toolkit” (Scottish & Southern Electricity Networks, 2019) • Balfour Beatty’s A Better Balance: a roadmap to BNG (Balfour Beatty 2018)
	Dec-Feb	First Defra consultation on Net Gain (Defra 2018c)	• Introduced the government’s proposed approach to BNG • Asked whether net gain should be mandated in the UK for developments in the scope of the Town and Country Planning Act (TCPA) (1990) • Suggests a “a 10% gain in biodiversity units would be a suitable level of net gain to require in order to provide a high degree of certainty that overall gains will be achieved, balanced against the need to ensure any costs to developers are proportionate” and that this “would be a mandatory national requirement, but should not be viewed as a cap on the aspirations of developers” (p. 30)
		Net Gain impact assessment published alongside consultation (Regulatory Policy Committee 2018)	• Recommended “net gain [be mandated] through the use of a specified biodiversity metric to development in scope of the Town and Country Planning Act, and adds a tariff component for compensation that cannot be delivered on the site or locally” (p. 1) • Emphasises the multiple objectives that had driven policy development were “that net gain: (1) delivers habitat creation, meeting the government's ambition to leave the environment in a better state than it inherited it; (2) is simple, streamlined and certain for developers, easy to understand and will not prevent, delay or reduce housebuilding; and (3) is of clear benefit to people and local communities” (p. 1) • 10% is chosen for amount of gain as “the lowest level of net gain that the department could confidently expect to deliver genuine net gain, or at least no net loss, of biodiversity and thereby meet its policy objectives” (p. 20) • Estimated BNG would have a direct cost £63.8 m per year (2017 prices), based on estimates of the additional cost of net gain delivery per ha of development and land developed for housing per year • Assumed only 10% of this (£6.4 m) would fall on developers, with assumptions that reductions in the value of land for development (associated with increased costs) would result in 90% of costs passing through to land prices, representing a loss of income for landowners • This represented 0.3% of the annual turnover of developers in England (£23.1bn) • *One of the first national policy impact assessments (if not the first) to quantify significant biodiversity/nature benefits, significant in securing political support for the policy*
2019	February	CIRIA, IEMA and CIEEM publish further BNG guidance for implementation of the good practice principles (Baker et al. 2019)	• Includes case studies and expansions on the original 2016 Good Practice Principles
		National Planning Policy Framework updated (Ministry of Housing, Communities and Local Government 2019)	• Wording on BNG does not change from 2019 version
	March	HM Treasury publishes Spring Statement (HM Treasury and Hammond 2019)	• Section on green growth includes the commitment that “the government will Mandate net gains for biodiversity on new developments in England to deliver an overall increase in biodiversity” (Clean growth bullet 2) • *Unusual for non-financial policy measures to be announced this way*
		Government commits to mandating BNG as part of the Environment Bill (Defra Press Office 2019)	• Gave confirmation that BNG would be part of the Environment Bill and, if passed, become part of English law
	July	Biodiversity Metric 2.0 is published as a beta test for consultation by Natural England (Crosher et al. 2019)	• *Intended to provide a standardised metric that could be used in place of the many organisational metrics that were being developed* • Addition of connectivity and strategic location for the calculation of base pre- and post- intervention units • Risk factor made up of difficulty of habitat creation x time to target condition x off-site risk also included for calculating post-intervention units • Addition of new “very high” distinctiveness score for highly threatened and internationally scarce habitats • Improved treatment of features such as urban trees and green roofs
		Summary of responses and government response to the first Defra consultation on Net Gain published (Defra 2019)	• Found that 78% of respondents supported mandatory net gain for developments in the scope of the TCPA • Some respondents highlighted issues such as planning authority capacity, presence of loopholes including the use of the tariff by developers to avoid responsibility, and focus on interests of developers over those of nature Committed to: o 10% net gain with no broad exemptions o support for Local Planning Authorities (LPAs) to address capacity issues o creation of a publicly available register of gains o exclusion of irreplaceable habitats o continued evaluation and minimisation on the impact on industry

#### Stage 5 (2019–2021): Tug-of-war via Parliament

During this period, biodiversity net gain is presented to Parliament as part of the Environment Bill. The EU-UK withdrawal agreement is renegotiated, removing the need for environmental non-regression. Biodiversity net gain legislation is debated in parliament, with motions to strengthen BNG legislation failing, with the government stating the motions would be infeasible or disproportionate. *The significant debate around the policy is likely compounded by significant lobbying both to strengthen the policy and, on the other hand, to ensure it does not significantly impact development*. Eventually, the Environment Act gains Royal Assent, creating a legal requirement to legislate for BNG. *Also within this period, corporate interest in biodiversity increases, including the rise of discussion around “Nature Positive”*. Key events in England during this period are shown in Table 5.

**Table 5:** Key events in Stage 5 (2019–2021) in which BNG is presented and debated within Parliament as part of the Environment Bill

Year	Month	Event	Relevance to BNG
2019	October	Final Defra impact assessment of BNG and local nature recovery strategies issued (Regulatory Policy Committee 2019b)	• Language around the tariff changed, instead stating that “[d]evelopers will have the option, once mitigation hierarchy has been demonstrated, to pay for the offset of remaining units through a biodiversity units market” (p. 1) however, the option of “payment to government who will provide statutory biodiversity credits into the compensation market” (p. 24) remained • Suggested considerably higher costs to developers £199 m per year, but again with 90% of this falling on landowners through changes to land prices • Included ongoing costs to local government of £9.5 m per year, which were not included in the previous impact assessment • Regulatory Policy Committee deemed impact assessment fit for purpose (Regulatory Policy Committee 2019a)
		Environment Bill 2019–19 (House of Commons 2019a) passes first and second readings in the House of Commons (Smith and Priestley 2020)	• Strengthened NERC (2006) general “duty to conserve biodiversity” to "duty to conserve and enhance biodiversity” • Required that the “biodiversity value attributable to the development exceeds the pre-development biodiversity value of the onsite habitat” (p. 206) by at least 10% • Covered developments under the TCPA (1990), excluding those permitted through development orders and urgent Crown development, making the submission and approval of a BNG plan a planning requirement • Provisions for the creation of “the biodiversity gains site register”, purchase of credits from the Secretary of State, requirement to publish a national habitat map for England, and conservation covenants • Included several clauses enabling the Secretary of State to propose secondary legislation to change BNG requirements after the bill becomes an Act of Parliament (known as Henry VIII clauses) • Published alongside explanatory notes (House of Commons 2019b) • Concern about lack of ambition within the wider bill, multiple ministers called for the bill to be strengthened to avoid regression from the UK’s high environmental standards under the EU
		UK Parliament net gain POST brief published (Wentworth 2019)	• Gives background on net gain for use by members of Parliament
		New EU-UK withdrawal agreement (Curtis et al. 2019)	• Removed need for environmental non-regression post- transition period
	November	Environment Bill 2019–19 falls at dissolution of Parliament (Smith and Priestley 2020)	• Paused legislative process for BNG until a future Parliament
	December	Intertidal habitats added to biodiversity metric calculator (Natural England 2019a, b)	• Allowed BNG to be applied to intertidal habitats in a more standardised manner
		Environment Bill 2019–20 announced in Queen’s speech (Prime Minister’s Office and Her Majesty The Queen 2019)	• Restarted legislative process for BNG
2020	January–February	Environment Bill 2019–20 passes first and second readings in House of Commons (Smith and Priestley 2020)	• Broadly the same as Environment Bill 2019–19 • Published alongside explanatory notes (House of Commons 2020) • Clarified that where sites already on the biodiversity gains site register are developed again, any further gain must be measured from the final intended metric value, irrespective of whether it had already been delivered • Concerns remained over non-regression from EU standards and the level of power afforded to the OEP
	February	The Biodiversity Metric 2.0 consultation closes (Natural England 2020)	• Summary and government response published in August • Allowed practical experience to be incorporated into the metric
	March	Biodiversity Net Gain: Financial & Economic Appraisal for Major Infrastructure Projects (WSP 2020)	• Commissioned by Defra • “[S]hows that the predicted costs of achieving 5%, 10% or 20% BNG outcomes for six major infrastructure projects is equivalent to around 1% of the capital costs of these schemes” (p. i)
	March–May	House of Commons Committee stage of Environment Bill 2019–20 (Smith 2021a) followed by Report Stage and Third Reading (Smith 2021b)	• Multiple Opposition amendments put forward to strengthen the protections afforded by the Bill • Calls to: make 10% a minimum that could only be revised upwards; secure gains in perpetuity; remove powers for Secretary of State to add to the list of exempted development; and strengthen OEP and its independence • All either failed on division or were withdrawn, with the Government arguing they were infeasible and disproportionate • *Ideas such as increasing the duration of protection for gains were also unpopular with many potential habitat providers* • Multiple Government amendments added limiting when the OEP can initiate an environmental review and initiate or intervene in judicial review proceedings
2021			
	February	Dasgupta review published (Dasgupta 2021)	• Presented research on treating nature as an economic asset, how to value biodiversity and how to treat nature as a portfolio with risk and uncertainty • Showed that acting for biodiversity now was more beneficial for the economy than delaying action and that the UK needed to do more to achieve a nature positive future, which would require conserving and improving nature, changing economic measures of success, and transforming institutions and systems
		Biodiversity Net Gain: Market analysis study published (eftec, WSP and ABPmer 2021)	• Commissioned by Defra • Recommendations included: o Increasing understanding of the BNG market o Minimising Government’s role as the seller of last resort o Promoting good mitigation hierarchy practice o Extending the BNG requirement to Infrastructure Projects o Investing in institutional capacity, training and transparency, both in terms of LPAs and independent oversight
		Defra Biodiversity Metric 3.0 and supporting information published (Panks et al. 2021)	• Removed connectivity from the metric • Was published with a small-sites metric, designed to make biodiversity assessments for small developments more proportionate • Included multiple other small improvements • *Created lots of interest from habitat providers*
	August	BS 8683—Process for designing and implementing Biodiversity Net Gain published (BSI 2021)	• Provided a framework to demonstrate that a project has followed a process based on UK-wide good practice • Aimed to help to avoid “greenwashing” claims around projects doing BNG
	June	Government response to Dasgupta Review (HM Treasury 2021)	• Government commits to “nature-positive” future in response to Dasgupta review • Announce intention to amend Environment Bill to include Nationally Significant Infrastructure Projects (NSIPs) within BNG *following a positive response to this within consultations*
	June–September	Environment Bill 2019–20 debated in House of Lords (Smith 2021b)	• Government amendment (Amendment 194B and new Schedule 14A/ Lords Amendments 55 and 93) includes NSIPs within BNG, significant as it requires BNG beyond the scope of the Town and Country Planning Act • Government amendment (Amendment 84/ Lords Amendment 91) requiring the Government to lay the new Biodiversity Metric and any amendments thereof before Parliament • Further Government amendment (Amendments 86 and 88/ Lords Amendments 57 and 92) to mean minimum duration of gains may only be increased from the 30 years initially tabled and for the potential for such an increase to be regularly reviewed (Ammendment 89/Lords Amendment 58) • Explanatory notes published prior to moving back to House of Commons (House of Commons 2021)
	September	“The State of No Net Loss/Net Gain and Biodiversity Offsetting Policy in English Local Planning Authorities” report published (Robertson 2021)	• Found that: o 56% of LPAs reported that it was currently practical to deliver biodiversity No Net Loss/ Net Gain o Resourcing was the main issue for LPAs that did not feel it was practical o 34% of LPAs already used some kind of metric in considering the ecological impact of planning applications o 32% of LPAs already had a mandatory No Net Loss/ Net Gain requirement for at least some planning authorities
	Oct-Nov	Environment Bill 2019–20 “ping pong” stages between Lords and Commons (Smith 2021b)	• All Government amendments made in the House of Lords were agreed and all non-Government amendments were disagreed or removed by further amendment • Disagreement about level of independence of the OEP • Lords eventually stopped insisting the OEP had full independence to carry out its functions as it saw fit, leaving substantial limits on OEP’s power
	November	Environment Act gains Royal Assent (Environment Act 2021)	• Set the requirement for development to deliver BNG in England, subject to its later commencement, and created powers to create regulations on the detail of the biodiversity net gain requirement

#### Stage 6 (2022 onwards): Implementation phase

This period represents the lead up to BNG coming into force including considerable consultation on and increased clarity about how BNG will be legislated for; increased funding for LPAs; and the publishing of guidance and the statutory tools. The official mandate is repeatedly delayed, *causing anger within some stakeholders*. Key events in England during this period are shown in Table 6.

**Table 6:** Key events in Stage 6 (2022 onwards) in which consultations and guidance precede BNG coming into force

Year	Month	Event	Relevance to BNG
2022	January-April	Defra consultation on Biodiversity Net Gain Regulations and Implementation (Defra 2022a)	• Consulted on proposed BNG regulations, notably specifics on: o Exemptions from BNG, including whether it is correct to not exempt brownfield sites and temporary developments o Exclusion of irreplaceable habitats from BNG o Last resort of purchasing statutory biodiversity credits from the UK Government where developers are demonstrably unable to achieve biodiversity net gain through on- and off-site options o Intent to mandate BNG for NSIPs by 2025 o Suggestion that developers could sell excess BNG units o The biodiversity gain site register, only for off-site gains o Allowing stacking of biodiversity units with other payments for environmental services, “provided they are paying for distinct, additional outcomes (for example, carbon sequestration and biodiversity benefits)” (p. 75) • Alongside this, the Government announced £4 million in funding for LPAs to prepare for mandatory BNG (Defra et al*.* 2022a, b)
	March	Joint open letter to Secretary of State for Levelling Up, Housing and Communities, Secretary of State for Environment, Food and Rural Affairs, and Chairman of Natural England (zu Ermgassen et al. 2022)	• Called for care to be taken that BNG fulfil its potential for nature recovery • Pointed out potential for BNG to allow loss of English nature if units promised fail to materialise • Highlighted three key issues for BNG to produce genuine gains: o Need for credible mechanisms for monitoring and enforcement of gains o Under-resourcing and skills deficit within local authorities, leading to limited oversight of BNG projects; and o Dominance of on-site gains as opposed to more ambitious and coordinated nature recovery efforts
	April	Defra Biodiversity Metric 3.1 and supporting information released (Panks et al. 2022)	• Relatively small changes from 3.0, mainly focussing on clarifying guidance and revising condition assessments (Natural England 2022)
	June	Defra consultation on marine net gain (Defra 2022b)	• Proposed looking at both habitats and species • Incorporation of environmental benefits conferred by biodiversity, while remaining “nature first” • Potential for a contributions-based rather than metric-based approach • Considered pressure reduction, as well as restoration • Will be mandatory
		OEP mission statement published (Office for Environmental Protection 2022)	• “[T]o protect and improve the environment by holding the government and other public authorities to account” (p. 5) • Confirmed the OEP would oversee LPAs, not be oversight for individual net gain projects
		Association of Local Government Ecologists (ALGE) publish results of Defra-funded survey looking at local authority capacity to carry out BNG (Snell and Oxford 2022)	• Found that LPAs are lacking the ecological capacity required for BNG • Only 5% of respondents felt they currently had adequate ecological resource to scrutinise all applications that might affect biodiversity • Fewer than 10% reported their current expertise and resources will be adequate to deliver BNG • Nearly half stated they do not regularly look at any advice or guidance
	July	Government response to joint open letter (Benyon 2022)	• Stated that work is being done on how to better enforce BNG and that the “Levelling Up and Regeneration Bill” will help to strengthen enforcement powers • Stated further funding for LPAs would be announced and changes to planning fees would also help with resourcing • Investigating inclusion of on-site gains in register • Future review of monitoring duration • Creating guidance about thresholds to be able to move to the next stage of the mitigation hierarchy
	Aug-Sept	Technical consultation on the biodiversity metric (Defra 2022c)	• Sought opinions on the metric prior to publishing the version that would likely become statutory
2023	January	Environmental Improvement Plan (update to 25 YEP required by Environment Act) (HM Government 2023a)	• Information on markets – publish policy framework in spring 2023 as part of updated Green Finance Strategy • 10% mandate to be introduced from November 2023 • Confirmed further funding would be available for LPAs • Mentioned exploring marine net gain • Cost recovery for environmental regulators
	February	Stacking guidance published (Defra and Natural England 2023)	• Confirmed stacking would be allowed with nutrients units • For voluntary schemes, e.g. carbon credits, only biodiversity units above what would have been created by standard practice for the voluntary credits can be claimed, e.g. further habitat enhancements that do not impact the carbon value
		Nationally Significant Infrastructure: action plan for reforms to the planning process published (Department for Levelling Up, Housing and Communities 2023)	• Sets November 2025 as the date from which BNG will be mandated for NSIPs • Confirms they will be subjected to the same 10% gain maintained for 30 years as other developments • Also confirms that marine net gain will be mandated, but does not give a date
		Government response to Defra consultation on BNG regulations and implementation (Defra 2023c)	• Confirmation of an extra £16.71 million of funding for LPAs to prepare for mandatory BNG • Defined the scope of BNG (i.e. what will be exempted) • Stated that secondary legislation on definitions of irreplaceable habitats will be added in future • Confirmed sale of “excess” on-site gains will be allowed • No centralised trading platform or recording of credit prices • No register for on-site gains, but investigating how to add information on on-site gains already within planning applications to the register • LPAs will be enforcing BNG, then they will be held accountable by OEP
	March	Defra Biodiversity Metric 4.0 and supporting information published (Natural England 2023b)	• Changes made primarily focused on ease of use (Natural England 2023a) • Also changes to spatial risk multiplier • Would likely form the basis of the statutory metric after being put before Parliament, expected to be in November 2023 (Burke 2023)
		Government response to consultation on the biodiversity metric (Defra 2023a)	• Will consider species inclusion for next metric update
		“Mobilising Green Investment” the Government Green Finance Strategy published (HM Government 2023b)	• Set target to “mobilise at least £500 million of private finance per year into nature’s recovery in England by 2027” (p. 74) citing BNG as a part of achieving this
		Summary of responses to Defra consultation on marine net gain published (Defra 2023f)	• Respondents highlighted need for ecosystem approach considering species and off-site impacts • 81% of respondents agreed Marine net gain should be mandatory
	May	Guidance for selling off-site units (Defra 2023d)	• Reiterates points made in previous documents
	July	UKHab 2.0 released	• Changes made to add new habitats and increase standardisation of use • Changes to codes mean not all habitats align with the previous UKHab 1.1
	September	BBC Report on “delays” to BNG (BBC News 2023)	• *Information about delays to BNG policy is leaked to the BBC*
		UK Government release updated timeline for BNG (Defra, Department for Levelling Up, Housing and Communities and Harrison 2023)	• Published later the same day as BBC report on delays • Moves expected date of mandate for most developments to January 2024 • Dates for other projects remain as April 2024 for small sites, and 2025 for Nationally Significant Infrastructure Projects • Commitment to publish the required guidance and regulations by the end of November
		Taskforce on Nature-related Financial Disclosures (TNFD) UK regional launch (TNFD 2024)	• Aim “to support a shift in global financial flows away from nature-negative outcomes and toward nature-positive outcomes”
	October	Levelling-up and Regeneration Act 2023 gains Royal Assent (Levelling-up and Regeneration Act 2023)	• Adds detail to the Town and Country Planning Act around the correct baseline to use in cases where the value of a habitat has been reduced prior to development
	November	Original expected date of mandatory BNG	• Three months before eventual mandate
		Government publish draft Statutory Metric and guidance (Defra 2023e)	• Draft Statutory metric has small updates from Defra metric 4.0 with updated guidance, including a very short list of irreplaceable habitats • Introduction of Biodiversity Gain Hierarchy, only requiring the mitigation hierarchy to be followed for habitats classified as “high” distinctiveness or higher, causing considerable controversy (Colley 2023)
	December	“Is England ready for biodiversity net gain?” Webinar (Rojo Martin 2023)	• Potential for draft guidance to change after concerns about Biodiversity Gain Hierarchy • Indicates there are likely to be changes to stacking guidance • Confirms early 2023 date for BNG mandate if “it’s not January, it will be 2 February, for instance”—Lucy Cheeseman, DEFRA deputy head of land use and head of net gain
		Government publish response to Marine Net Gain consultation (Defra 2023b)	• Confirms inclusion of both biodiversity and wider environmental benefits and use of both active and pressure reduction interventions • States the Government will continue working on an assessment framework and run proof of concept projects
2024	January	Rescheduled expected date of BNG mandate (Defra, Department for Levelling Up, Housing and Communities and Harrison, 2023; Vaughan 2024)	• Expected date of mandate delayed to February 2024 for major developments and April for small sites
	February	BNG mandated for major developments of February 12th (Fisher 2024)	• Date from which “large” developments within the scope of the Town and Country Planning Act will be required to demonstrate a 10% biodiversity net gain to get planning permission • State that guidance has been updated based on stakeholder comments
	April	BNG mandated for small sites (Gowers 2024)	• Small sites within the scope of the Town and Country Planning Act are required to demonstrate a 10% biodiversity net gain to begin work from April 2nd
2025	November	Expected introduction of BNG mandate for Nationally Significant Infrastructure Projects (Defra, Department for Levelling Up, Housing and Communities and Harrison 2023)	• Date from which NSIPs are expected to be subject to mandatory BNG

## Reflections from the authors

As well as documenting the events that led to BNG in England, this paper represents an unusual opportunity to gather the reflections of a wide range of expert's views. In doing so, however, it is important to note that the views within this section are not shared by all authors. Rather the paper attempts to draw together our views so they can be used by others working on BNG or related legislation and policy. The authors broadly agree that the effects of BNG have been mixed. For better outcomes, more consistent standards and enforcement are needed (especially for on-site and off-site delivery) and more capacity is needed for their application, particularly in local authorities.

Most of the authors of this paper have watched and/or actively driven BNG as it evolved from early pilots of biodiversity offsetting, through voluntary implementation of BNG, and on to its current mandatory state under the amended Town and Country Planning Act 1990. Strongly competing pulls of ambition and pragmatism have been present throughout this timeline. There are many examples of this, for example in the inclusion of connectivity as a qualitative element of BNG assessments rather than in the metric (reflecting a view that the quantitative GIS tool was not fit for purpose for nationwide release as part of the statutory metric at that time, though it could form part of future metric updates) and in the (lack of) inclusion of species impacts in the biodiversity metric which, although part of BNG assessments and design, was seen by some as essential and by others as too complex.

For some authors, the move to BNG has been associated with a shift towards biodiversity being seen as an asset, rather than a liability or risk, and has catalysed a greater emphasis on steps to avoid harm. The development of BNG based on voluntary practice by industry based on the groundwork laid by the biodiversity offsetting scoping studies and pilots and combining it with best internationally developed practice principles was seen as a strength, requiring a considerable level of collaboration over a decade to develop the tools and legislation as we see them today. Further, these authors see the legislation as having true potential for nature recovery and enhancement at a local level, particularly in combination with Local Nature Recovery Strategies.  It has been influential in the development of a nature market to channel much-needed private investment into nature recovery, although this should not be seen as circumventing necessary government regulation.

Other authors, view BNG is a positive and powerful way of thinking, in principle, but feel that it can be perceived or used as a tool to enable development in practice. Where the policy aims to address the incremental biodiversity loss across England, they feel that the current implementation is not sufficiently comprehensive. The metric uses habitat as a proxy for biodiversity. As such, it is a “good start” for measuring and comparing biodiversity impact, but does not cover all important aspects of a habitat, for instance it insufficiently considers outcomes for species populations or indirect and cumulative impacts on biodiversity. However, one author considered that outcomes for species populations and indirect and cumulative impacts are addressed under different laws, and the 10% BNG is additional to mitigation and compensation already provided, there was dispute between authors as to whether this adequately addressed these impacts. Further, there is evidence that much of the compensation promised under BNG, particularly on-site, is unrealistic (see Rampling et al. 2024). There is substantial variation in the implementation of BNG between local planning authorities (LPAs) at present. While some LPAs are incredibly thorough with BNG, others do not have the resources or expertise to sufficiently check the design of proposed Habitat Management and Monitoring Plans (HMMPs) and ensure compliance with them, potentially resulting in inadequate and low-quality habitat delivery. This also reflects the need to build the skills of ecologists following the policy shift from a species-focused approach to legislation to long-term habitat restoration, enhancement, and creation.

## Conclusions

This timeline represents an important step in documenting the inception and evolution of BNG policy in England. This timeline has two main uses: the first is a source of learning for countries and institutions looking to implement similar policies, and the second is as a starting point and collection of documents for analyses of biodiversity net gain in England. As BNG practice develops and issues inevitably arise, as with all policies, we hope this timeline will be used to understand the root of such issues, thus helping develop solutions. We believe the timeline also has many other potential uses, such as: a starting point to better understand how BNG interacts with other English policies and the emerging concept of “Nature Positive”; in the future research on the changing value given to, and language used for biodiversity in English policy; and understanding political undercurrents that have driven the path of events seen in this timeline. It is only with such research we can create an understanding of what policies like BNG are likely to mean for nature in the context of their accelerating adoption globally.

## Abbreviations

ALGE: Association of Local Government Ecologists
BBOP: Business and Biodiversity Offsets Programme
BDO: Biodiversity Offsetting
BNG: Biodiversity Net Gain
Brexit: The United Kingdom's withdrawal from the European Union (EU)
Defra: Department for Environment and Rural Affairs
EU: European Union
HS2: High Speed (Rail) Two
IEMA: Institute of Environmental Management and Assessment (renamed ISEP Institute of Sustainability & Environmental Professionals in July 2025)
JNCC: Joint Nature Conservation Committee
LPA: Local Planning Authority
OEP: Office for Environmental Protection
NSIP: Nationally Significant Infrastructure Projects
TNFD: Taskforce on Nature-related Financial Disclosures
UKHab: UK Habitat Classification
25 YEP: 25 Year Environment Plan
MEA: Millenium Ecosystem Assessment
NNL: No net loss
NERC (2006): Natural Environment and Rural Communities Act (2006)
TCPA (1990): Town and Country Planning Act (1990)
CIEEM: Chartered Institute of Ecology and Environmental Management
CIRIA: Construction Industry Research and Information Association
IEMA: Institute of Environmental Management and Assessment
UN: United Nations
UK NEA: UK National Ecosystem Assessment
UK BAP: UK Biodiversity Action Plan
